# Heterologous Expression of *Aedes aegypti* Cation Chloride Cotransporter 2 (aeCCC2) in *Xenopus laevis* Oocytes Induces an Enigmatic Na^+^/Li^+^ Conductance

**DOI:** 10.3390/insects10030071

**Published:** 2019-03-14

**Authors:** Megha Kalsi, Christopher Gillen, Peter M. Piermarini

**Affiliations:** 1 Department of Entomology, The Ohio State University, Ohio Agricultural Research and Development Center, Wooster, OH 44691, USA; kalsi.5@osu.edu; 2 Department of Biology, Kenyon College, Gambier, OH 43022 USA; gillenc@kenyon.edu

**Keywords:** *Xenopus* oocyte, mosquitoes, cation chloride cotransporters, two-electrode voltage clamping

## Abstract

The yellow fever mosquito *Aedes aegypti* possesses three genes encoding putative Na^+^-coupled cation chloride cotransporters (CCCs): aeNKCC1, aeCCC2, and aeCCC3. To date, none of the aeCCCs have been functionally characterized. Here we expressed aeCCC2 heterologously in *Xenopus* oocytes and measured the uptake of Li^+^ (a tracer for Na^+^) and Rb^+^ (a tracer for K^+^). Compared to control (H_2_O-injected) oocytes, the aeCCC2-expressing oocytes exhibited significantly greater uptake of Li^+^, but not Rb^+^. However, the uptake of Li^+^ was neither Cl^−^-dependent nor inhibited by thiazide, loop diuretics, or amiloride, suggesting unconventional CCC activity. To determine if the Li^+^-uptake was mediated by a conductive pathway, we performed two-electrode voltage clamping (TEVC) on the oocytes. The aeCCC2 oocytes were characterized by an enhanced conductance for Li^+^ and Na^+^, but not K^+^, compared to control oocytes. It remains to be determined whether aeCCC2 directly mediates the Na^+^/Li^+^ conductance or whether heterologous expression of aeCCC2 stimulates an endogenous cation channel in the oocyte plasma membrane.

## 1. Introduction

The sodium potassium chloride cotransporters (NKCCs) belong to the solute carrier-12 (SLC-12) superfamily of cation chloride cotransporters (CCCs), which also includes sodium chloride cotransporters (NCCs) and potassium chloride cotransporters (KCCs) [1]. Typically, the CCCs cotransport cations and chloride in an electroneutral fashion and are inhibited by loop diuretics (e.g., furosemide, bumetanide) and/or thiazide. [2]. CCCs play critical roles in a wide variety of physiological functions in vertebrates, including neuronal development, regulation of cell volume, and maintenance of intracellular Cl^−^ concentration [3,4,5,6,7,8].

In insects, CCCs have not been as extensively studied, but pharmacological and genetic studies in fruit flies and mosquitoes suggest critical roles in neuronal function and transepithelial fluid secretion [9,10,11,12,13,14,15]. Previously, we characterized the expression of genes encoding putative Na-coupled CCCs in the yellow fever mosquito *Aedes aegypti* [16]. The genome of *Ae. aegypti* contains three putative Na-coupled CCCs, which we have designated as aeNKCC1 (also known as aeCCC1), aeCCC2, and aeCCC3. In larval and adult female *Ae. aegypti*, aeNKCC1 and aeCCC2 mRNAs were highly expressed in the Malpighian tubules and hindgut, whereas aeCCC3 mRNAs were primarily expressed in the anal papillae of larvae [16]. The aeNKCC1 gene contains substantial similarity to vertebrate bumetanide-sensitive NKCCs and *Drosophila melanogaster* NCC69 (CG4357), a bonafide bumetanide-sensitive NKCC that contributes to transepithelial fluid secretion in Malpighian tubules [13,14,15]. On the other hand, aeCCC2 and aeCCC3 are part of an insect-specific clade of putative NKCCs. The third membrane-spanning domain (TM3) of both aeCCC2 and aeCCC3 contains large sequence divergence from aeNKCC1, suggesting potential novel transport properties and/or pharmacology of the CCC2/3 clade from aeNKCC1 [16]. Consistent with this notion, a *Drosophila* ortholog of aeCCC2/3 (CG31547) expressed in High Five cells was found to not enhance Rb^+^ influx [15], which potentially indicates a lack of K^+^ transport.

The objective of the current study was to heterologously express aeCCC2 in *Xenopus laevis* oocytes and characterize its functional and pharmacological properties. We found that expressing aeCCC2 in oocytes promoted an influx of Li^+^ (a tracer for Na^+^), but not of Rb^+^ (a tracer for K^+^), suggesting potential for Na^+^, Cl^−^ cotransport. However, the Li^+^ uptake was independent of extracellular Cl^−^ and insensitive to thiazide and loop diuretics. Two-electrode voltage clamping of the aeCCC2-expressing oocytes revealed an enhanced conductive pathway for Na^+^ and Li^+^, but not K^+^, compared to H_2_O-injected oocytes. Our results suggest that heterologous expression of aeCCC2 stimulates an endogenous cation channel in the oocyte plasma membrane and/or that aeCCC2 possesses channel-like properties independent of conventional CCC activity.

## 2. Materials and Methods

### 2.1. Cloning and cRNA Preparation

The aeCCC2 cDNA (AAEL009888) was synthesized de novo and cloned into a pGH19 plasmid by GENEWIZ (South Plainfield, NJ, USA). The aeCCC2 cDNA plasmid was digested using the restriction enzyme NotI, and the capped RNA (cRNA) was synthesized using the mMessage mMachine T7 Transcription Kit (Thermo Fisher Scientific, Waltham, MA, USA). Further, the cRNA was purified using an RNeasy MinElute Cleanup Kit (Qiagen, Hilden, Germany) according to manufacturer’s protocol and dissolved in the nuclease-free water.

### 2.2. Heterologous Expression in *Xenopus laevis* Oocytes

Defolliculated *Xenopus* oocytes were purchased from Ecocyte Bioscience (Austin, TX, USA). Oocytes were injected with 82.8 nL of CCC2 cRNA (1 ng/nL) dissolved in nuclease-free water using a Nanoject II microinjector (Drummond Scientific, Broomall, PA, USA). Control oocytes were injected with the same volume of nuclease-free water. After injections, the oocytes were incubated in standard Barth’s saline (SBS; Ecocyte Bioscience, Austin, TX, USA) for 3 days at 18 °C. The SBS buffer consisted of NaCl (88 mM), KCl (1 mM), CaCl_2_ (0.4 mM), Ca (NO_3_)_2_ (0.33 mM), MgSO_4_ (0.8 mM), NaHCO_3_ (2.4 mM), sodium pyruvate (0.275 g/L), penicillin (1000 U/mL), and streptomycin (0.1 mg/mL).

### 2.3. Measurement of Ion Uptake in *Xenopus* Oocytes

Similar to Durr et al. [17], we used a non-radioactive approach to measure Li^+^ and Rb^+^ uptake in *Xenopus* oocytes. We used Li^+^ as a tracer for Na^+^, and Rb^+^ as a tracer for K^+^. All of the following steps took place in the wells of a 24-well tissue culture plate (Falcon™, Thermo Fisher Scientific, Waltham, MA, USA) at 25 °C unless indicated otherwise. In brief, 10–15 oocytes were transferred from SBS to ND96 (see Table 1 for composition) to rinse off the SBS. After 30 min, the oocytes were transferred to a low sodium and low chloride isotonic pre-uptake buffer (buffer I, Table 1) for 30 min. Previous studies have found that low chloride incubation promotes NKCC activity [18,19,20,21]. The oocytes were then transferred to an isotonic uptake buffer containing 25 mM LiCl and 25 mM RbCl (buffer II), 25 mM LiCl (buffer III), 25 mM Li-gluconate (buffer IV), or 25 mM Li-iodide (buffer V) for 45 min (Table 1). For experiments testing pharmacological inhibitors, the pre-uptake and uptake buffers contained the inhibitors at a final concentration of 100 μM or 1 mM (0.1% DMSO). All pre-uptake and uptake buffers contained 0.5 mM ouabain to inhibit the activity of the endogenous Na, K-ATPase.

After the uptake period, the oocytes were washed three times (5 min each) in ice-cold buffer I. Following the third wash, each oocyte was added to a separate 1.5-mL microcentrifuge tube containing 250 μL of ultrapure water (MilliQ, Millipore, Burlington, MA, USA) and frozen at −80 °C. Each oocyte was lysed by thawing the tube at room temperature and pipetting the oocyte through the orifice of a 200-μL pipette tip several times. The oocyte lysate was transferred to a 2-mL polypropylene tube with 0.45-µm cellulose acetate filter (Corning Costar Spin-X, Corning, NY, USA) and centrifuged for one minute at 13,000 g to remove cell debris and yolk particles.

Cation concentrations of filtered oocyte lysates were evaluated using a Dionex 500-DX-chromatography system (Sunnyvale, CA, USA) that consisted of a CS12a cation-exchange column, CSRS-Ultra 4-mm suppressor, AS50 autosampler with 25-μL injection loop, ED40 conductivity detector, and Chromeleon 6.7 software (Thermo Fisher) [22]. Samples were eluted with 18 mM methanesulfonic acid (MSA) for 15 min at a flow rate of 1 mL/min. Cations were quantified by comparison to a standard curve of certified check standards (Sigma Chemical; Spex Certiprep, Metuchen, NJ, USA). An anion exchange column was not available and therefore measurements of anions in the samples were not possible.

### 2.4. Western Blots of Total Membrane Protein

Total membrane fractions were prepared from aeCCC2 and H_2_O-injected oocytes 3 days after injection using a previous protocol [23]. In brief, the oocytes were lysed with a plastic pestle in ND96 buffer diluted 10× with dH_2_O and supplemented with protease inhibitors (Halt™ protease inhibitor cocktail and 0.5 M EDTA; Pierce, Thermo-Fisher Scientific). The crude lysate was transferred to a 1.5-mL tube and centrifuged at 3000 *g* for 10 min at 4 °C. The supernatant was then transferred to open top ultracentrifuge tubes (Seton Scientific, Petaluma, CA, USA) and centrifuged for 1.5 h at 100,000× *g* at 4 °C in a Beckman ultracentrifuge (Beckman Coulter, Brea, CA, USA). The membrane pellets were resuspended in the lysis buffer and total protein was determined with a bicinchoninic acid assay (Pierce Biotechnology, Rockford, IL, USA). Following total protein determination, an equal volume of a 2× urea buffer was added to the sample followed by 5× Laemmli sample buffer [24].

The samples were heated to 90 °C for 5 min and separated on a denaturing 8% polyacrylamide gel for molecular mass separation. The separated proteins were transferred to a polyvinylidene difluoride (PVDF) membrane (Thermo Fisher Scientific) and blocked with 5% nonfat dry milk dissolved in Tween-Tris-buffered saline (TTBS) as described previously [25]. A rabbit anti-aeCCC2 affinity-purified polyclonal antibody from a previous study was used [16]. The blocked PVDF was treated with the primary anti-aeCCC2 antibody (1:500 ratio in blocking buffer) overnight at 4 °C. The PVDF membrane was then washed with TTBS at room temperature three times (5 min each) and incubated with horseradish peroxidase (HRP)-conjugated goat anti-rabbit secondary antibody (1:15,000 ratio in blocking buffer) for 90 min at room temperature. Immunoreactivity was detected using an enhanced chemiluminescence kit (SuperSignal™ West Pico PLUS Chemiluminescent Substrate, Pierce Biotechnology, Waltham, MA, USA). The chemiluminescent signal was captured digitally using a MyECL Imager (Model 62236X; Thermo Fisher Scientific).

### 2.5. *Xenopus* Oocyte Electrophysiology

We used two-electrode voltage clamping (TEVC) to measure the electrophysiological properties of *Xenopus* oocytes as described previously [23]. During an experiment, the oocyte was bathed under ND96 solution (4 mL/min) in a RC-3Z chamber (Warner Instruments, Hamden, CT, USA) and impaled with two KCl filled electrodes. Resting membrane potential was allowed to stabilize for approximately 1–2 min, followed by V_m_ clamping to a hyperpolarizing potential of 30 mV relative to the resting V_m_ to promote inward membrane currents (I_m_). In some experiments, the membrane potential (V_m_) in ND96 was recorded and the oocytes were subjected to a voltage-stepping protocol to measure the current (I)–voltage (V) relationships. In other experiments, the solution was switched to a low Na^+^ and K^+^ buffer (buffer VI, Table 1) and the V_m_ was clamped to 30 mV more negative than the resting V_m_ to continuously record I_m_ and promote inward I_m_. The solution was then switched to a buffer containing 25 mM K^+^ (buffer VII), Na^+^ (buffer VIII), or Li^+^ (buffer IX) for ~90 s to observe effects on I_m_.

### 2.6. Statistics

For the statistical analysis, GraphPad Prism software (version 7.0 d) was used. Comparisons between two groups were performed using unpaired *t*-tests (*p* < 0.05); comparisons among more than two groups were performed using one-way ANOVAs (*p* < 0.05).

## 3. Results and Discussion

### 3.1. Heterologous Expression of aeCCC2 in *Xenopus* Oocytes

**Figure 1:**
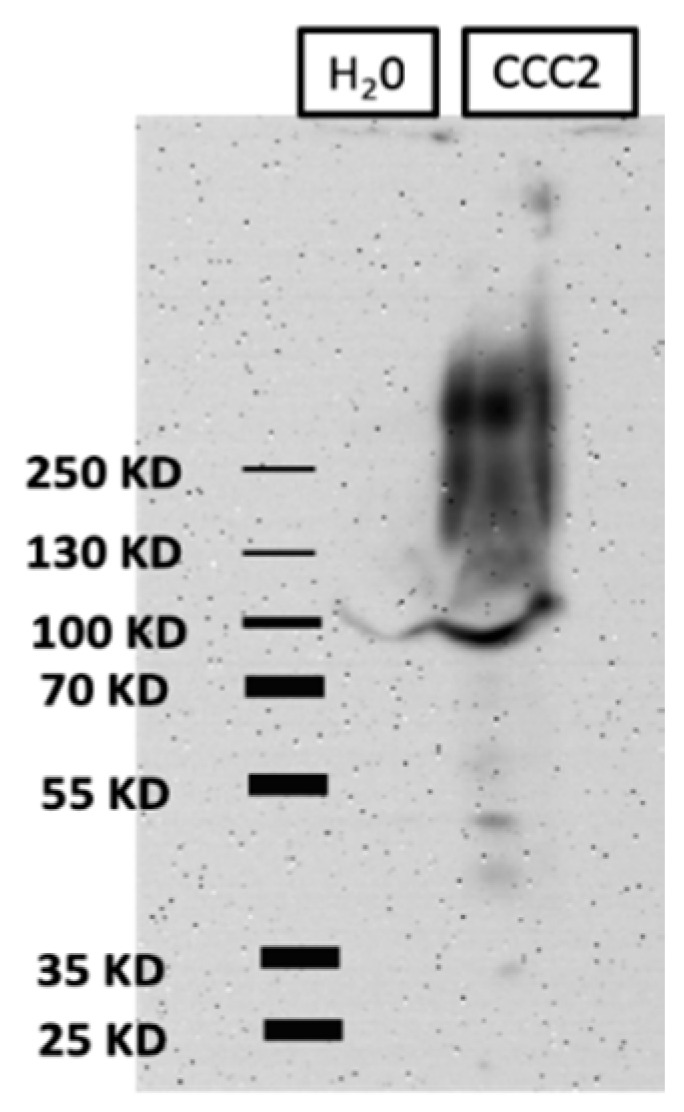
Heterologous expression of aeCCC2 in *Xenopus* oocytes. Western blot of total membrane fractions prepared from oocytes 3 days after injection with aeCCC2 cRNA (80 ng) or nuclease-free H_2_O (80 nL). Molecular mass markers (kDa) are on the left. Note that an immunoreactive band of ~100 kDa appeared in membrane fractions of both H_2_O-injected and aeCCC2 oocytes, which presumably indicates reaction of the aeCCC2 antibody with an endogenous protein in *Xenopus* oocytes.

Western blotting of oocyte membrane fractions revealed broad aeCCC2-immunoreactivity that ranged from just over 100 kDa to over 250 kDa in aeCCC2 oocytes and was not present in H_2_O-injected oocytes (Figure 1). The lower range of the broad immunoreactivity is consistent with the predicted size of 117 kDa for the aeCCC2 monomer, whereas the higher range of immunoreactivity is consistent with the presence of glycosylated forms. There are three predicted *N*-linked glycosylation sites on the fourth extracellular loop of the deduced aeCCC2 protein. We have previously shown that the analogous extracellular loop of another mosquito CCC (aeKCC1) is heavily glycosylated when expressed in *Xenopus* oocytes and that its glycosylation promotes functional activity [25]. Thus, the western blotting results suggest that the aeCCC2 protein is expressed and post-translationally modified by *Xenopus* oocytes.

### 3.2. Functional Characterization of aeCCC2

**Figure 2:**
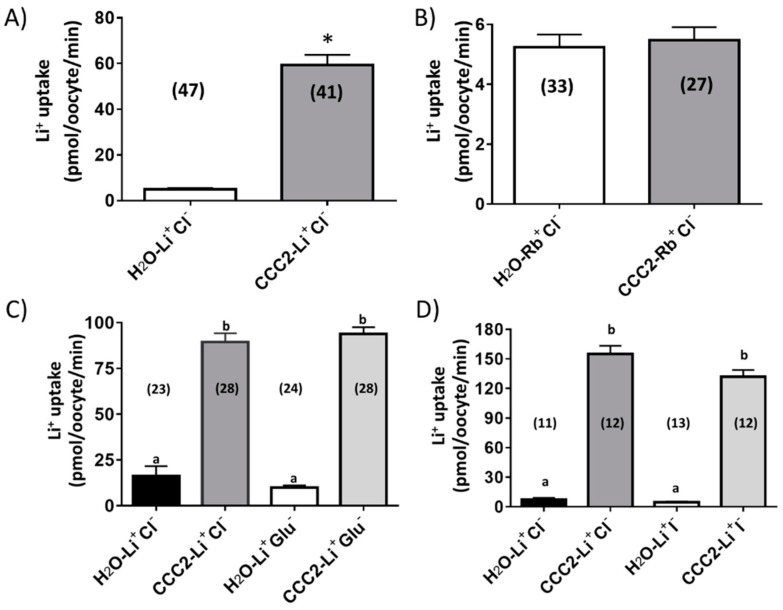
Rates of Li^+^ (**A**) and Rb^+^ (**B**) uptake measured in *Xenopus* oocytes three days after injection of aeCCC2 cRNA (80 ng) or nuclease-free H_2_O (80 nL). The * represents a significant difference using a Student’s *t*-test (*p* < 0.05). Effects of substituting extracellular Cl^−^ with gluconate (**C**) or iodide (**D**) on Li^+^ uptake rates in aeCCC2 or H_2_O-injected oocytes. The lowercase letters indicate statistical categorization of the means as determined by an ANOVA with a Tukey post-test (*p < 0.05*). In all panels, values are means ± SEM based on the number of oocytes in parentheses.

As shown in Figure 2A, aeCCC2 oocytes were characterized by a significantly greater uptake of Li^+^ (~7-fold) compared to H_2_O-injected oocytes. In contrast, the uptake of Rb^+^ was similar between aeCCC2 and H_2_O-injected oocytes (Figure 2B). These data suggest that expression of aeCCC2 in *Xenopus* oocytes increased membrane permeability to Na^+^ but not K^+^. The Rb^+^ uptake data are consistent with those of Sun et al. [15] who found that *Drosophila* CCC2 (CG31547) expression did not enhance Rb^+^ uptake when expressed in High Five cells. (Li^+^ uptake was not measured by Sun and colleagues).

**Figure 3:**
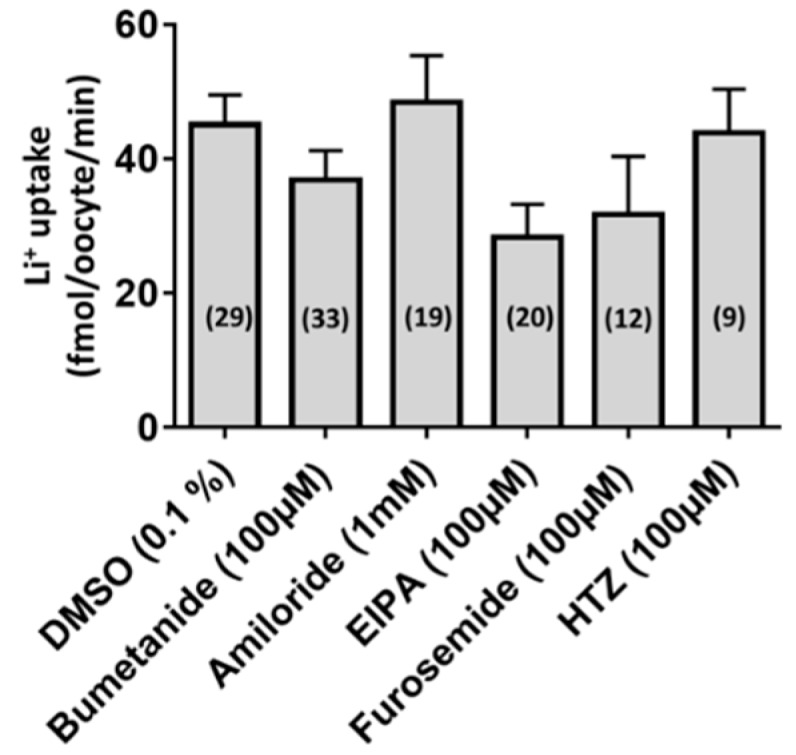
Pharmacology of the Li^+^ uptake in *Xenopus* oocytes heterologously expressing aeCCC2. Rates of Li^+^ uptake was measured in *Xenopus* oocytes 2–3 days after injection with aeCCC2 cRNA (80 ng) or H_2_O (80 nL). Inhibitor concentrations are indicated on the x-axis. DMSO (0.1%) served as the control. Values are means ± SEM based on the number of oocytes in parentheses. A one-way ANOVA was performed, which was not significant (*p > 0.05*). EIPA: ethyl isopropyl amiloride; HTZ: hydrochlorothiazide.

To determine if the Li^+^ uptake in aeCCC2 oocytes was dependent on extracellular Cl^−^, we replaced it with iodide (LiI) or gluconate (Li-gluconate). To our surprise, aeCCC2 oocytes had significantly greater Li^+^ uptake than H_2_O-injected oocytes in the presence or absence of extracellular Cl^−^, regardless of substitution with gluconate or iodide (Figure 2C,D). We also tested whether the uptake of Li^+^ in aeCCC2 oocytes was affected by common inhibitors of (1) CCCs (bumetanide, furosemide, hydrochlorothiazide: HCTZ); (2) Na/H exchangers (ethyl isopropyl amiloride: EIPA); or (3) other Na^+^-transporters/channels (amiloride). When compared with the uptake of Li^+^ in oocytes treated with DMSO (the vehicle for the inhibitors), none of the inhibitors significantly inhibited the influx of Li^+^ (Figure 3). The results of Figure 2; Figure 3 are consistent with the notion that aeCCC2 may have different transport properties and pharmacological sensitives compared to NKCCs [16]. Moreover, these results suggest that the uptake of Li^+^ in aeCCC2 oocytes was not mediated by endogenous EIPA/amiloride-sensitive Na/H exchangers and/or Na-transporters/channels.

### 3.3. Electrophysiological Characterization of aeCCC2 in the *Xenopus* Oocytes

**Figure 4:**
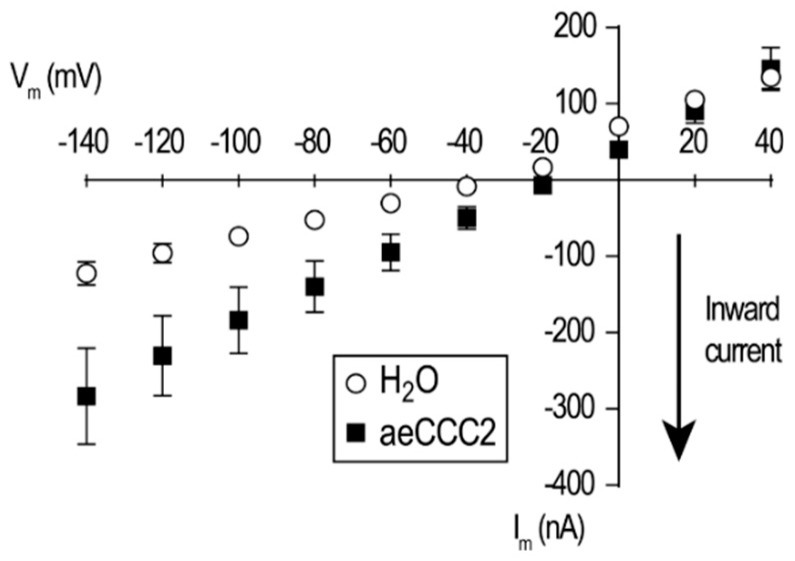
The mean current–voltage (I–V) relationships of *Xenopus* oocytes in ND96 2–3 days after injection with aeCCC2 cRNA (80 ng) or H_2_O (80 nL) as determined by TEVC. Values are means ± SEM; *N* = 3 oocytes each for aeCCC2 and H_2_O.

Given the unexpected transport properties and pharmacology of the aeCCC2 oocytes in the ion uptake assays, we next examined their electrophysiological properties using TEVC. When bathed in ND96, the resting membrane potential (V_m_) of aeCCC2 oocytes (−26.03 ± 2.8 mV; *N* = 3 oocytes) was significantly depolarized (*p* < 0.05; *t*-test) compared to that of H_2_O-injected oocytes (−48.06 ± 3.2 mV; *N* = 5 oocytes). In addition, the I–V relationships of the aeCCC2 oocytes in ND96 revealed greater inward currents across all hyperpolarizing voltages examined compared to H_2_O-injected oocytes (Figure 4). Thus, the heterologous expression of aeCCC2 appears to activate an inward-rectifying conductance in the oocyte membrane.

**Figure 5:**
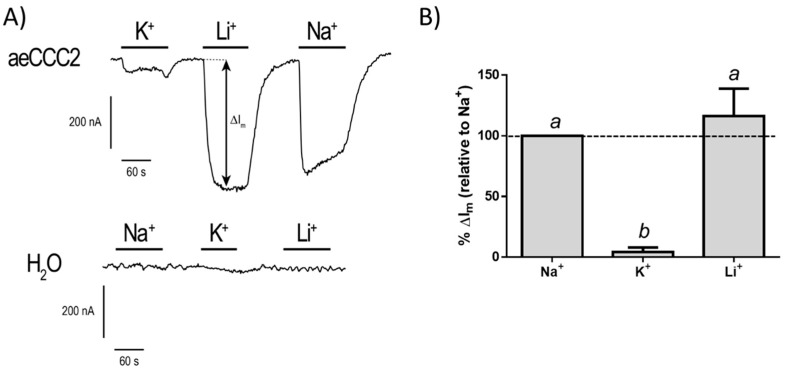
(**A**) Representative tracings of membrane current (I_m_) in aeCCC2 or H_2_O-injected oocytes that were clamped at −85 mV and −100 mV, respectively. The bidirectional arrows signify the change in membrane current (ΔI_m_); (**B**) Summary of relative effects of Na^+^, K^+^, and Li^+^ on the ΔI_m_ in aeCCC2 oocytes. For each oocyte and cation, the ΔI_m_ values were normalized to the ΔI_m_ elicited by Na^+^ and corrected for the average ΔI_m_ in H_2_O-injected oocytes (*N* = 5) associated with that cation. Values are means ± SEM, based on four aeCCC2 oocytes. Lowercase letters indicate statistical categorization of the means based on a one-way repeated-measures ANOVA and Tukey’s post-test (*p < 0.05*).

To characterize the cation selectivity of the conductance in aeCCC2 oocytes, we measured the I_m_ in oocytes (voltage-clamped at a hyperpolarizing V_m_) and increased the concentration of extracellular Na^+^, Li^+^, or K^+^ to 25 mM. As shown in Figure 5A, when K^+^ was increased there was a small change of I_m_ (ΔI_m_) in the aeCCC2 oocytes, whereas there was a relatively large ΔI_m_ when the Na^+^ or Li^+^ concentration of the bath was elevated. In H_2_O-injected oocytes, similar changes to the extracellular cation concentrations produced nominal changes to I_m_ (Figure 5A). Figure 5B summarizes the changes in ΔI_m_ produced by each cation in the aeCCC2 oocytes normalized to that produced by Na^+^. In brief, the ΔI_m_ produced for Na^+^ and Li^+^ were similar to each other, whereas that produced by K^+^ was ~25-times lower than Na^+^. Thus, the cation selectivity sequence of the conductance in aeCCC2 oocytes was Na^+^ = Li^+^ > K^+^. Preliminary attempts to block the Na^+^ conductance with 0.1 mM or 1 mM gadolinium (GdCl_3_), a generic blocker of non-selective cation channels, or 1 mM amiloride, a generic blocker of Na channels and transporters, were without detectable effects. Thus, our results suggest that heterologous expression of aeCCC2 in *Xenopus* oocytes promoted a Na^+^/Li^+^ conductance in the plasma membrane, which was presumably responsible for the enhanced Li^+^ uptake observed in aeCCC2 oocytes (Figure 2A). Based on the cation-selectivity sequence of this conductance, it most closely resembles that previously described by Nessler et al. [26] who found that heterologous expression of a *Plasmodium falciparum* chloroquine resistance transporter in *Xenopus* oocytes stimulated the expression of an endogenous non-selective cation conductance (G_cat_) that was more permeable to Na^+^ and Li^+^ relative to K^+^.

Collectively, the results of the present study suggest that aeCCC2 may not function as a typical CCC. In the literature, there are a few cases where CCCs have been reported to possess physiological roles independent of their transport activity. For example, the *Xenopus laevis* NKCC1 was found to be responsible for induction of the embryo’s secondary axis independent of Cl^−^ [27]. Moreover, in mice, the neuron-specific role of KCC2 in dendritic spinogenesis or the development of the central nervous system was independent of its ion transport function [28,29]. Thus, it is possible that aeCCC2 is a structural or signaling protein and through its interactions with endogenous oocyte proteins leads to the activation of an endogenous Na^+^/Li^+^ conductance. Alternatively, aeCCC2 might secondarily activate an endogenous Na^+^/Li^+^ conductance in *Xenopus* oocytes if it transports a solute that we have not measured. NH_4_^+^ is one possibility, because it can substitute for K^+^ on vertebrate NKCCs [30,31].

We also cannot rule out that aeCCC2 is in part directly responsible for the Na^+^/Li^+^ conductance. There are some documented cases of transmembrane proteins acting as both a transporter and channel, such as excitatory amino acid transporters (EAATs), which can function as glutamate transporters and Cl^−^ channels [32,33]. Moreover, an electroneutral Na-bicarbonate cotransporter has been shown to carry a Na^+^ current [34]. Further, some transporters have evolved into modified ion channels, such as the cystic fibrosis transmembrane conductance regulator (CFTR), which is the only ATP-binding cassette (ABC) protein that functions as a Cl^−^ channel [35]. Additional electrophysiological studies will be required to fully characterize the transport properties of aeCCC2 and the unexpected Na^+^/Li^+^ conductance in the aeCCC2 oocytes.

An intriguing question raised by our results is whether aeCCC2 directly or indirectly mediates Na^+^-conductances in the plasma membranes of mosquito Malpighian tubules and the hindgut where its mRNA is found [16]. In particular, based on our results in *Xenopus* oocytes, it is tempting to speculate that aeCCC2 contributes to Na^+^-conductances in the basolateral membrane of principal cells in Malpighian tubules of adult females. Under resting conditions, amiloride-insensitive Na^+^-channels account for ~15% of the basolateral membrane conductance in principal cells [36]. If the Malpighian tubules are treated with the calcitonin-like peptide (also known as diuretic hormone 31, DH_31_) or cyclic adenosine monophosphate (cAMP; the second message of DH_31_), then an amiloride-sensitive Na^+^ conductance is activated [36,37,38,39]. This DH_31_/cAMP-induced Na^+^-conductance promotes a highly selective natriuresis in Malpighian tubules, which helps rid the mosquito hemolymph of excess NaCl and water after a blood meal [40,41,42,43,44,45].

To date, the molecular identities of the amiloride-insensitive and cAMP-stimulated Na^+^ conductances in the basolateral membranes of Malpighian tubules have not been determined. The functional and pharmacological properties of the aeCCC2 oocytes in the present study, which were characterized in the absence of cAMP, are consistent with the former Na^+^ conductance. Moreover, we cannot rule out that aeCCC2 contributes to the latter Na^+^ conductance. For example, it is possible that cAMP modulates the activity and amiloride-sensitivity of the Na^+^-conductance in aeCCC2 oocytes. Notably, the deduced aeCCC2 amino acid sequence contains predicted protein kinase A (PKA) acceptor sites with the sequence R-X-T/S, suggesting that aeCCC2 is regulated by changes in intracellular cAMP (via PKA). Future studies should test this hypothesis in *Xenopus* oocytes and determine the membrane localization of aeCCC2 protein in mosquito Malpighian tubules.

## 4. Conclusions

Here we heterologously expressed aeCCC2 in *Xenopus* oocytes. Ion uptake and electrophysiological experiments suggested that aeCCC2 may not transport the typical substrates of CCCs and may have channel-like properties. Future studies will be required to confirm such unconventional roles/characteristics of aeCCC2 and elucidate its physiological roles in mosquitoes.

## Figures and Tables

**Table 1:** Chemical composition of buffers used in *Xenopus* oocyte ion uptake and electrophysiology experiments. All values in the table are in mM. Osmolality of the buffers was confirmed to be 195 ± 5 mOsmol/kg H_2_O using a vapor pressure osmometer (Wescor, Logan, UT, USA). The pH of all buffers was adjusted to 7.5 with NaOH, NMDG, HCl, or gluconic acid depending on the dominant cation and presence/absence of Cl^−^ in the buffer. Ouabain (0.5 mM) was added to all buffers except ND96. NMDG: N-methyl-d-glucammonium; Gluc: gluconate; HEPES: (4-(2-hydroxyethyl)-1-piperazineethanesulfonic acid).

Buffer	ND96	I	II	III	IV	V	VI	VII	VIII	IX	Manufacturer
NaCl	96						0.5	0.5	25	0.5	Thermo Fisher Scientific
KCl	2						0.5	25	0.5	0.5	Thermo Fisher Scientific
LiCl		25	25							25	Acros Organics (Waltham, MA, USA)
RbCl			25								Acros Organics
MgCl_2_	1	1	1				1	1	1	1	Thermo Fisher Scientific
CaCl_2_	1.8	1.8	1.8				1.8	1.8	1.8	1.8	Thermo Fisher Scientific
K-Gluc				2							Acros Organics
Li-Gluc						25					BOC Sciences (Shirley, NY, USA)
LiI					25						Thermo Fisher Scientific
Mg-Gluc				1	1	1					Sigma-Aldrich (St. Louis, MO, USA)
Ca-Gluc				1.8	1.8	1.8					Sigma-Aldrich
NMDG		75	50	48	75	75	97.5	75	75	75	Acros Organics
Sucrose				77							Thermo Fisher Scientific
HEPES	5	5	5	5	5	5	5	5	5	5	Thermo Fisher Scientific
